# Visualizing the Distribution of Matrix Metalloproteinases in Ischemic Brain Using In Vivo ^19^F-Magnetic Resonance Spectroscopic Imaging

**DOI:** 10.1155/2019/8908943

**Published:** 2019-01-06

**Authors:** Vincent J. Huber, Hironaka Igarashi, Satoshi Ueki, Mika Terumitsu-Tsujita, Chikako Nito, Ken Ohno, Yuji Suzuki, Kosuke Itoh, Ingrid L. Kwee, Tsutomu Nakada

**Affiliations:** ^1^Center for Integrated Human Brain Science, Brain Research Institute, University of Niigata, Niigata, Japan; ^2^Administrative Section of Radiation Protection, National Institute of Neuroscience, National Center of Neurology and Psychiatry, Tokyo, Japan; ^3^Department of Neurological Science, Graduate School of Medicine, Nippon Medical School, Tokyo, Japan; ^4^Department of Neurology, University of California Davis, Davis, CA, USA

## Abstract

Matrix metalloproteinases (MMPs) damage the neurovascular unit, promote the blood-brain barrier (BBB) disruption following ischemic stroke, and play essential roles in hemorrhagic transformation (HT), which is one of the most severe side effects of thrombolytic therapy. However, no biomarkers have presently been identified that can be used to track changes in the distribution of MMPs in the brain. Here, we developed a new ^19^F-molecular ligand, TGF-019, for visualizing the distribution of MMPs in vivo using ^19^F-magnetic resonance spectroscopic imaging (^19^F-MRSI). We demonstrated TGF-019 has sufficient sensitivity for the specific MMPs suspected in evoking HT during ischemic stroke, i.e., MMP2, MMP9, and MMP3. We then utilized it to assess those MMPs at 22 to 24 hours after experimental focal cerebral ischemia on MMP2-null mice, as well as wild-type mice with and without the systemic administration of the recombinant tissue plasminogen activator (rt-PA). The ^19^F-MRSI of TGN-019-administered mice showed high signal intensity within ischemic lesions that correlated with total MMP2 and MMP9 activity, which was confirmed by zymographic analysis of ischemic tissues. Based on the results of this study, ^19^F-MRSI following TGN-019 administration can be used to assess potential therapeutic strategies for ischemic stroke.

## 1. Introduction

Matrix metalloproteinases (MMPs) are essential to normal brain function [1–3]; however, they can become highly toxic to the brain in pathological situations, such as cerebral ischemia, where they evoke degradation in tissue integrity via the neuroinflammatory cascade. This cascade eventually leads to cerebral edema and hemorrhagic transformation (HT), a life-threatening complication of cerebral ischemia [4]. Moreover, MMP inhibition was shown to ameliorate tissue damage and preserve the blood-brain barrier in animal ischemia models [5]. Three in MMP family members, MMP2, MMP9 (referred to as the gelatinases), and MMP3, are thought to be activated within the ischemic lesion where they are critical to injury progression during and after cerebral ischemia [6, 7]. Of these MMPs, the concentration of MMP9 rises significantly in ischemic tissue consequent to thrombolytic therapy using the tissue plasminogen activator (tPA), the current gold-standard for treating acute stage ischemia. The rise of MMP9 was found to strongly correlate with increased risk of HT [8].

Visualization of MMP distribution in vivo would not only provide insights into tissue disintegration within the ischemic lesion but also enable the prediction of deleterious edema and HT. Some in vivo applicable techniques were developed for this specific purpose [9, 10], and however, they generally require relatively long-waiting times (ca. 4 to 24 hours) to reveal postadministration MMP distribution. Consequently, an in vivo method for visualizing MMP distribution during the acute stage of cerebral ischemia immediately following tracer administration would be extremely valuable. In response to that need, we developed the small molecule ^19^F-magnetic resonance (MR) ligand TGF-019 based on the broad-spectrum MMP inhibitor galardin (GM-6001), which has high affinity for MMP2, 3, and 9 [11]. TGF-019 visualization utilized the in vivo ^19^F-MR method first developed by Nakada, which has an advantage in the lack of ^19^F-background due to fluorinated compounds in the brain [12–14]. Using TGF-019, we visualized MMP distribution under three relevant sets of conditions using a mouse model of focal cerebral ischemia and correlated the average TGF-019 signal intensities in the brain region and individual MMP levels by the conventional methods following brain extraction.

## 2. Methods

### 2.1. Synthesis of TGF-019

**Figure 1:**
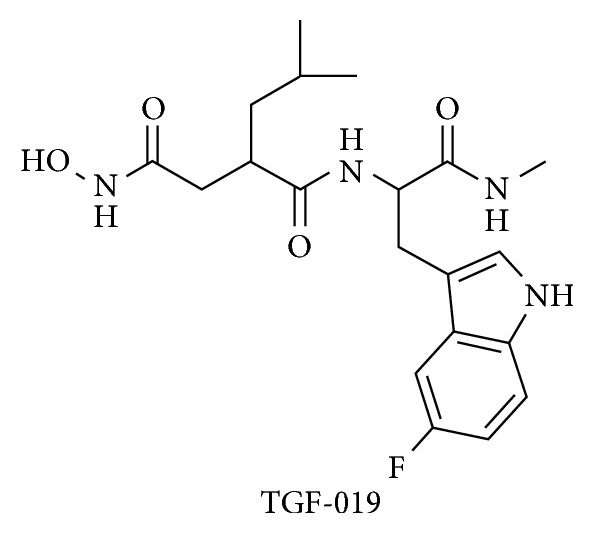
Chemical structure of TGF-019. Synthetic methods were shown schematically in Figure S1 and described in the supplemental materials section of the online version of this paper.

TGF-019 (Figure 1) was synthesized as a diastereotopic mixture starting from racemic(5-fluoro)tryptophan, shown schematically in Figure S1 and as described in detail in the supplementary materials section of this paper (Supporting Material). (Rac)-2-[(ethoxycarbonyl)methyl]-4-methylpentanoic acid was obtained from American Biochemicals (College Station, TX, USA) and was used as received. Additional reagents were sourced from Sigma-Aldrich (Tokyo, Japan), Wako Pure Chemical Industries (Osaka, Japan), TCI (Tokyo, Japan), or Nacalai Tesque (Kyoto, Japan) and were used as received. Analytical thin-layer chromatography (TLC) was performed using Sigma-Aldrich F254 indicating TLC plates, which were visualized under UV light, unless otherwise noted. ^1^H-nuclear magnetic resonance (NMR) spectra were recorded at 300 MHz on a Varian Mercury 300 spectrometer (Varian Inc, Palo Alto, CA, USA) and were referenced to an internal tetramethylsilane (TMS) standard, unless otherwise indicated. Analytical ultraperformance liquid chromatography (UPLC) and high-resolution mass spectroscopy (HRMS) were performed on a Waters (Milford, MA, USA) Acquity UPLC combined with a Waters LCT Premier XE mass detector, with additional UPLC data obtained using Waters Acquity UPLC PDA and ELS detectors.

The chemical properties of TGF-019 and its key intermediates were in agreement with those expected based on the assigned structures and were found to be similar to those described for nonfluorinated analogs [15]. UPLC analysis of the synthesized TGF-019 indicated it was obtained in 95% chemical purity as a 2.4 : 1 mixture of unresolved diastereomers. The product composition was further confirmed by HRMS analysis of the diastereomeric mixture (mass calculated for C_20_H_28_FN_4_O_4_ (M+H^+^), 407.2089; found, 407.2074 (3.6 ppm)). Prior to in vivo studies, a sample of TGF-019 was converted to its sodium salt by treatment with an equimolar amount of NaOH in ultrapure water, which was then lyophilized to give a powdered material suitable for reconstitution into an appropriate vehicle. No degradation in compound purity of the sodium salt was observed by UPLC/HRMS.

### 2.2. In Vitro MMP Inhibition

The ability of TGF-019 to interact with MMP2, MMP3, and MMP9 was evaluated using an in vitro inhibition assay (Supporting Material). In this assay, outsourced to Eurofins Panlabs Discovery Services (Taipei, Taiwan), TGF-019 was shown to completely inhibit MMP2 and MMP9, with a somewhat weaker, but significant inhibition of MMP3. This suggests increased MMP expression following ischemic injury can be visualized using TGF-019.

### 2.3. Animal Preparation

The study was approved by the institutional animal care and use committee of University of Niigata (61-6, 121-3) and carried out in accordance with the guidelines set forth by the U.S. National Institutes of Health regarding the care and use of animals for experimental procedures. MMP2 knockout mice [16] (RBRC00398) were provided by RIKEN BRC through the National Bio-Resource Project of the MEXT, Japan. Twenty-two adult male mice, C57BL/6, and twenty-one MMP2 knockout mice (26–30 g each) were maintained under standard laboratory conditions with a 12 h/12 h light/dark cycle. Food and water were available ad libitum, except for 10 h prior to MCA occlusion, during which food was withheld to prevent hyperglycemia.

### 2.4. Intracranial MMP Administration

To evaluate the sensitivity of TGF-019 to MMP2, MMP3, and MMP9, we injected each MMP individually into the cerebral hemisphere of MMP2 KO mice (*n*=2/MMP) [17] and imaged using ^19^F-MRSI following TGN-019 administration. MMP2 KO mice were chosen to avoid the background signal of baseline MMP2 in the normal brain. Under urethane anesthesia (1.2 g/kg intraperitoneal injection), the head of the mouse was fixed in a stereotaxic device, and 2 *μ*l of 0.1 *μ*g/*μ*l pro-MMP2, MMP3, pro-MMP9, or activated-MMP9 was injected 0.1 mm anterior to the bregma, 1.5 to 2 mm lateral to the midline, and 1 to 3.5 mm below the skull surface using a 30-gauge Hamilton syringe. The injection was performed over 15 min and was followed by MR measurement. Activation of pro-MMP9 was performed according to the method of Collier et al. [18].

### 2.5. Experimental Ischemic Stroke Models

Thirteen MMP2 knockout mice (the MMP2 KO group), eleven C57BL/6 mice with saline administration (the WT saline group), and eleven C57/BL6 mice with rt-PA administration (the t-PA group) were employed for the experimental ischemic stroke model. Mice were anesthetized with 1–1.2% isoflurane in 30% oxygen and 70% nitrous oxide administered through a face mask, with the animals breathing spontaneously. Rectal temperature was maintained at 37 ± 0.5°C using a temperature control unit and heating pad during the anesthesia period. Oxygen saturation (SpO2) was monitored throughout the operation procedure utilizing a pulse oximeter Mouse Ox (STARR Life Sciences Co, Oakmont, PA, USA) with probe placement on the left thigh. Regional cerebral blood flow (rCBF) was measured continuously starting immediately prior to and throughout the 60 min interval of induced focal ischemia using laser-Doppler flowmetry (ALF21, Advance Co., Tokyo, Japan). The Doppler probe was affixed to the skull 1 mm posterior and 6 mm laterally to the bregma. C57BL/6 animals received a continuous i.v. infusion of r-tPA, 10 mg/kg, dissolved in 0.3 ml normal saline for 30 minutes starting 15 minutes before recirculation. Control animals were given an identical volume of saline.

Transient focal cerebral ischemia was induced using a modified version of the procedure [19] described by Yang et al. [20]. The right middle cerebral artery (MCA) was occluded by introducing a 6-0 silicone-coated monofilament into the internal carotid artery to a point 6 mm distal to the internal carotid artery and pterygopalatine arterial bifurcation. Success of the MCA occlusion was confirmed in mice fulfilling the following three conditions: greater than 93% of SpO_2_ throughout the operation procedure, greater than 80% decrease in rCBF (CBF%) at 15–20 min following the ischemic insult relative to the preischemia level, and a neurological deficit score of 2 or greater at 30 min after ischemia after allowing the mouse to regain consciousness. Neurological deficit scoring was done using the criteria established by Amiry-Moghaddam et al. [21]: normal motor function, 0; flexion of the torso and contralateral forelimb upon lifting the animal by the tail, 1; circling to the contralateral side but normal posture at rest, 2; leaning to the contralateral side at rest, 3; and no spontaneous motor activity, 4. The filament was removed under anesthesia 60 min after ischemia induction.

### 2.6. In Vivo ^19^F-MRSI Acquisition and Data Analysis

Under anesthesia with an intraperitoneal administration of urethane (1.2 g/kg), 100 mg/kg TGF-019 dissolved in 0.2 ml saline was administered for about 30 seconds through tail vain 1 h following intracranial MMP administration or at 22 to 24 h after the induction of ischemia. Mice were then placed into the MR magnet on their backs in a custom-made Plexiglas stereotactic holder. The head was fixed in a position by ear and tooth bars. Rectal temperature was maintained at 37 ± 0.5°C using a custom-designed temperature control system.

MRI experiments were performed on a 15 cm bore 7T horizontal magnet (Magnex Scientific, Abingdon, UK) with a Agilent Unity-INOVA-300 system (Agilent Inc., Palo Alto, CA, USA) equipped with an actively shielded gradient. A custom-made volume transmit and quadrature surface receive ^1^H-^19^F double tune coil (Takashima Seisaku-Syo, Hino, Japan) was used for both ^1^H-anatomical MRI and ^19^F-MRSI. After acquisition of a ^1^H-T_2_-weighted image (TR/effective TE; 2000/80 m·sec, echo train; 8, echo spacing; 20 ms, field of view; 16 × 16 mm, image matrix 128 × 128, slice thickness; 2 mm), ^19^F-spin echo MRSI was acquired starting at 30 min post-TGF-019 administration using the following parameter settings: TR/TE; 1000/2.5 m·sec, field of view 16 × 16 mm, image matrix 16 × 16, spectral width 19841 Hz/2048 point, slice thickness 4 mm, number of acquisitions 16, and total scan time 68 min.

Data were processed using conventional 3D-FT. For chemical shift dimension, 40 Hz of exponential apodization was applied as a noise filter to both sides of the echo signals. For spatial dimensions, sine bell apodization was applied to avoid intervoxel signal contamination, and the matrix was zero filled to a 64 × 64 matrix. Spatial resolution was then enhanced to a 128 × 128 matrix by cubic interpolation. The MRSI map was coregistrated on to T_2_-weighted image, and the average signal intensity of TGF-019 within the infarcted area is shown by T_2_-weighted imaging, as well the contralateral hemisphere was measured to compare signal intensities among all three groups. Data processing was done using VNMR3.2 software (Agilent Inc., Palo Alto, CA, USA).

### 2.7. Assay of MMPs

Fifteen mice (five mice in each group) subjected to experimental ischemic stroke were used for the assay of MMPs. Under deep anesthesia using 200 mg/kg pentobarbital, the brain was extracted, and each hemisphere was cut 2 mm posterior from the frontal apex in 4 mm thick slices. Tissues were homogenized in the working buffer (50 mM Tris-HCl (pH 7.4), 1 *µ*M ZnCl_2_, 5 mM CaCl_2_, and 0.05% Brij35) [22]. Gelatin zymography was used to evaluate the activities of MMP2 and MMP9 according to the method described by Yang et al. [23] with some modifications. The gels were scanned on a densitometer (SH-9000, Corona, Ibaraki, Japan). A mixture of human MMP9 and MMP2 (Primary Cell Co. Ltd. Sapporo, Japan) served as gelatinase standards. MMP3 was determined by ELISA using an anti-MMP3 antibody (LF-EK50675 Mouse MMP-3, Ab frontier, Seoul, Korea).

### 2.8. Statistical Procedure

Statistics were performed with SPSS software (IBM corp., Armonk, NY, USA). Data were tested with one-way analysis of variance with the Bonferroni correction for multiple comparison. Data were expressed as mean ± standard error of the mean (SEM). Significance was considered where *P* < 0.05.

## 3. Results

### 3.1. Sensitivity of TGN-019 against MMPs

**Figure 2:**
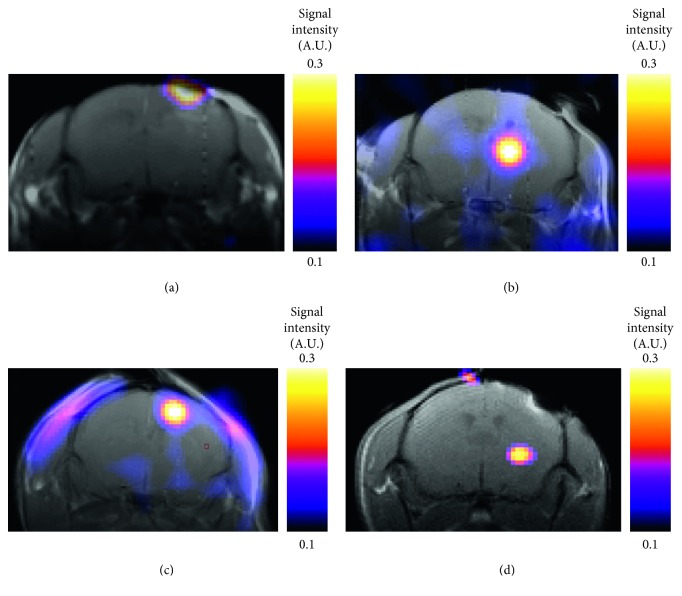
^19^F-MRSI of MMPs intracranially administered in the MMP2 knockout mice brain. MRSI color images were coregistered and superimposed with T_2_-weighted ^1^H-MRI. (a) Pro-MMP9, (b) activated MMP9, (c) pro-MMP2, or (d) pro-MMP3 was administered at 30 min before intravenous administration of TGF-019.

In the brains administrated with a microdose (approimately 0.2 *μ*g) of pro-MMP2, MMP3, pro-MMP9, or activated MMP9, ^19^F-signals corresponding to TGF-019 were clearly detected 30 min following a 200 mg/kg intravenous administration of the ligand (Figure 2). All eight mice survived the procedure.

### 3.2. Visualizing the Distribution of MMPs in Ischemic Stroke

**Figure 3:**
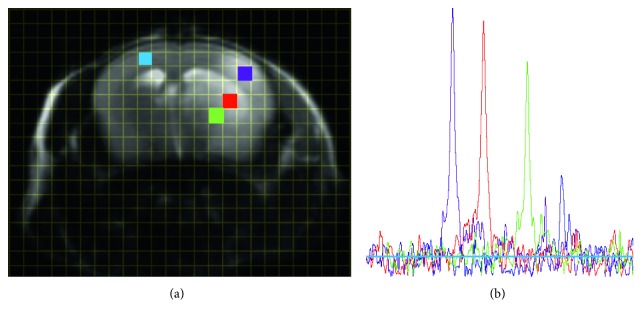
^19^F MRSI spectra in regions of interest. (a) T2-weighted image of representative t-PA-administered mouse shows the prominent infarct area in the right MCA area. Grid shows phase-encoded voxels in nonzero-filled/interpolated MRSI data. Each colored square corresponds to the voxel of region-of-interest (ROI) in which (b) the spectra of identical color was detected.

**Figure 4:**
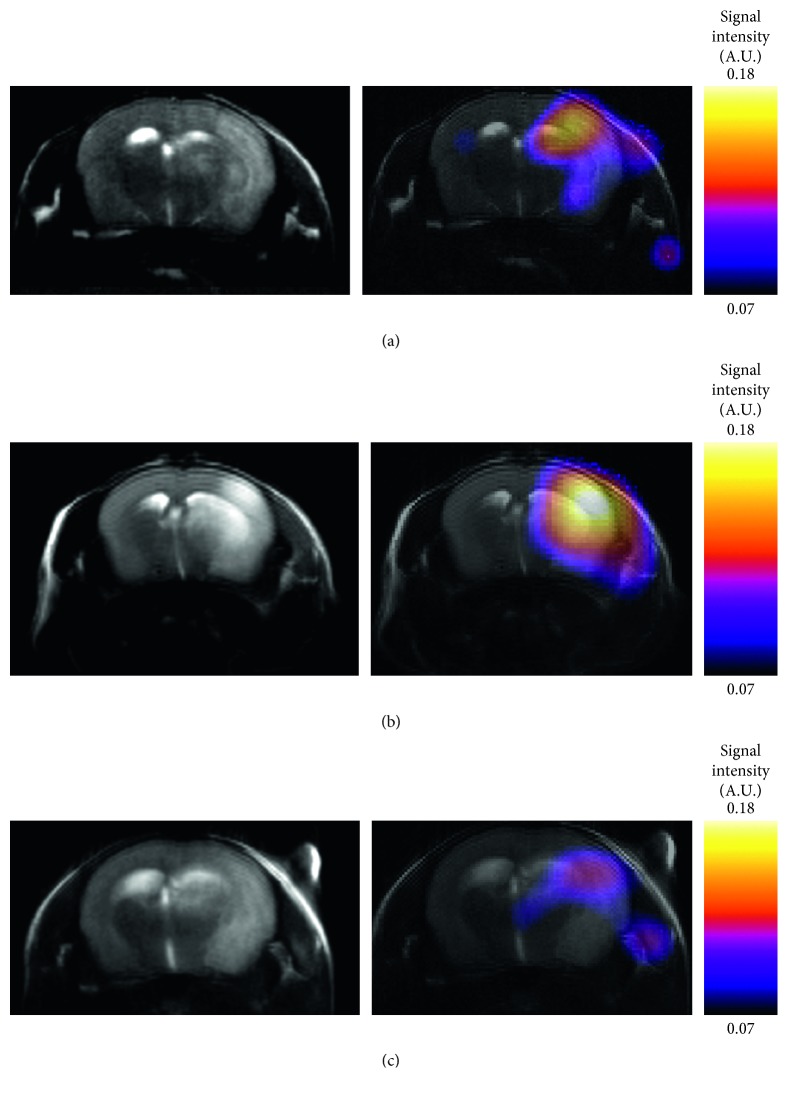
Representative MR images of the mice ischemia model. T_2_-weighted images (the left column) and ^19^F-MRSI coregistered and superimposed with T_2_-weighted images (the right column) of ischemia with intravenous administration of the saline (WT-saline) group (a), ischemia with intravenous administration of t-PA (t-PA) group (b), and MMP2 knockout (MMP2 KO) group (c).

**Figure 5:**
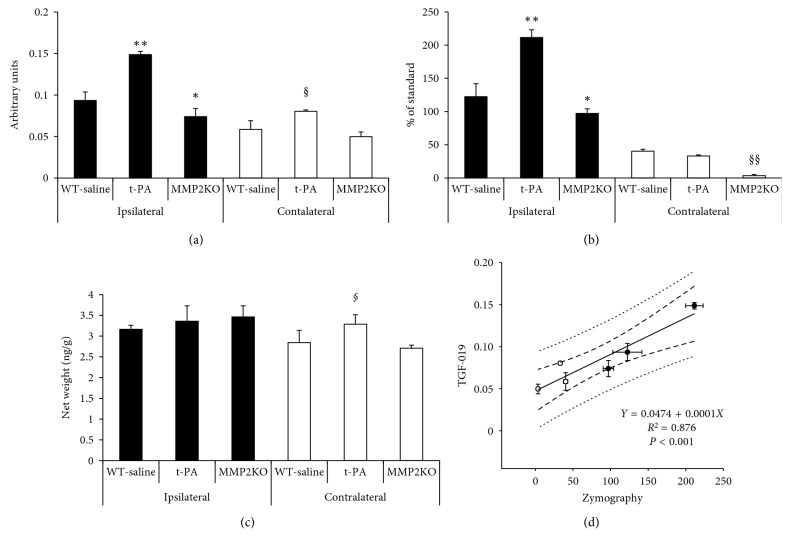
Changes of MMPs at 22 to 24 hours after 60 minutes of ischemia. (a) TGF-019 signal intensities in the ischemic area (ipsilateral, closed bar) and nonischemic (contralateral, open bar) area. *n*=4 per group. (b) MMP2 and MMP9 combined activity (MMP2/9) demonstrated by zymography. t-PA groups showed the strongest activity in the ischemic area. *n*=5 per group. (c) Protein levels of MMP3 did not show the significant changes among ischemic lesions. *n*=5 per group. (d) Signal intensities of TGN-019 and activities of MMP9/2 demonstrated by zymography were highly correlated in the range showed in the experiments. ^*∗*^ *P* < 0.05 vs ischemic area of the WT-saline group, ^*∗∗*^ *P* < 0.01 vs ischemic area of the WT-saline group, §*P* < 0.05 vs nonischemic area of the WT-saline group, §§*P* < 0.01 vs nonischemic area of the WT-saline group.

Following the MCA occlusion, nine mice in each of the C57BL/6 and MMP2 KO groups survived throughout the experiments. Infarcted tissue was indicated in the right MCA perfused area of all twelve mice studied by MR. The signal intensity of TGF-019 within the ischemic lesion delineated by T_2_-weighted imaging was significantly stronger than the nonischemic contralateral hemisphere in sufficient signal-to-noise ratio (23.7 in ischemic cortex, Figure 3). Of those groups, TGF-019 signal intensities were strongest in the t-PA group followed by the WT-saline group, while those of the MMP2 KO group were found to be the weakest (Figure 4 and Figure 5(a), *p* < 0.05, ANOVA with Bonferroni correction). Optical zymographic densities also showed significantly higher densities in the ischemic hemisphere than the contralateral hemisphere (Figure 5(b)). Conversely, MMP3 did not show a significant increase among all areas in this experimental procedure(Figure 5(c)). Signal intensities of TGN-019 and zymographic optical densities of MMP2/9 were found to be highly correlated in the range shown by the experiments (Figure 5(d)).

## 4. Discussion

TGF-019 is fluorinated derivative of pan-MMP inhibitor galardin (GM-6001), which has a high affinity for MMP2, MMP9, and MMP3 (Ki ≅ 0.5, 0.2, and 30 nM, resp.) [24], and penetrates the blood-brain barrier into the extracellular space of the brain parenchyma where MMPs exist and mainly play a physiological role in extracellular matrix regulation [25]. Physiologically, the basal distribution of MMPs consists of pro-MMP2, MMP3, and MMP14 (MT1-MMP) within extracellular space, while only a small amount of other MMPs are thought to be present [26]. Given the high affinity of galardin for those MMPs (MMP14 Ki ≅ 13.4 nM), TGF-019 signal intensity in normal brain tissue likely reflects its interaction with background levels of pro-MMP2, MMP3, and MMP14.

In the acute ischemic lesion, TGF-019 images showed significant MMP upregulation, and the resulting signal intensities correlated with the total proteolytic activity of MMP2 and MMP9. In particular, levels of activated MMP9 drastically increase during acute stage cerebral ischemia, which is pivotal in the cascade leading to neurovascular unit injury, especially deterioration of the blood-brain barrier [27]. Furthermore, tPA treatment further increased TGF-019 signal intensity within the ischemic lesion concomitant with increased MMP2/9 proteolytic activity, which was previously reported [8]. MMP2 is also upregulated in the ischemic lesion, and however, only moderate levels of MMP2 induction were reported in experimental cerebral ischemia [28]. While MMP3 also appears to be activated during acute stage ischemia and is also thought to contribute to neurovascular unit injury [29], no increase in its level were found in the three groups included in this study, which was also consistent with a prior report involving temporally MCA occluded mice [7]. One study did show a significant rise in MMP3 protein levels 24 h after the induction of cerebral ischemia [30], and however, the study involved a photochemically-induced permanent ischemia model, which may be responsible for the differences in MMP3 expression compared to the present study. Therefore, based on the currently available evidence, we believe the rise in TGF-019 signal intensity in our animal model primarily reflects MMP9 induction and upregulation within the ischemic lesion, although we should note that TGF-019 MRS would not discriminate MMP9 among other MMP family members.

Using TGF-019 as a potent tracer to visualize the distribution of MMP family members focusing on in vivo and future clinical studies, there are two main issues to be solved. One is the potential toxicity of TGF-019. In this study, we did not notice any behavioral changes after 100 mg/kg TGF-019 administration in MMP-treated mice, although we did not evaluate long-term behavioral or pathological changes. It was reported that administration of another broad-spectrum MMP family inhibitor, BB-94, evoked increased apoptosis in a cerebral hemorrhage model of mice [31]. However, GM-6001 at 180 mg/kg single dose, which is about twice the dose of TGF-019 used in the present study, did not show adverse effects and improved locomotor activities at seven days after induction of MCA occlusion [25]. Another issue is the relatively high dose (100 mg/kg) used in this study. Since we could detect the TGF-019 spectra with a high signal-to nose ratio (S/N ≅ 7 in normal tissue, S/N ≅ 21 in the ischemic lesion, resp., Figure 3) from small voxel (4 *μ*m^3^), it will be feasible to utilize TGF-019 at smaller doses than 100 mg/kg for a large object.

## 5. Conclusion

In summary, we synthesized TGF-019, a fluorinated compound with high-affinity to several MMP family members, and visualized their distribution in vivo using ^19^F-MRSI. Signal intensities of TGF-019 correlated to MMP2/9 activities, which were measured by zymography. While further studies will be necessary to confirm the relative contributions of the individual TGF-019 stereoisomers during ^19^F-MRSI, ^19^F-MRSI following TGN-019 administration can be used to elucidate the pathological role of MMPs during cerebral ischemia and to assess potential therapeutic strategies for ischemic stroke.

## Data Availability

The imaging data and numerical data used to support the findings of this study are available from the corresponding author upon request.

## Supplementary Materials

Supplementary Materials The supplementary material represents synthesis and in vitro MMP inhibition of TGF-019.
