# Supplementary material for: Visualizing the Distribution of Matrix Metalloproteinases in Ischemic Brain Using In Vivo 19F-Magnetic Resonance Spectroscopic Imaging
Source: Contrast Media Mol Imaging. 2019 Jan 6;2019:8908943. doi: 10.1155/2019/8908943 (PMC6339703; doi:10.1155/2019/8908943)
Supplement: Supplementary Materials — The supplementary material represents synthesis and in vitro MMP inhibition of TGF-019. [file 8908943.f1.pdf]

Supplemental Materials

Visualizing the distribution of Matrix metalloproteinases in ischemic brain using in vivo ^{19}F-MRSI

Vincent J Huber[1], Hironaka Igarashi[1], Satoshi Ueki[1], Mika Terumitsu-Tsujita[2], Chikako Nito[3],

Ken Ohno[1], Yuji Suzuki[1], Kosuke Itoh[1], Ingrid L Kwee[4], Tsutomu Nakada[1, 4]

[1] Center for Integrated Human Brain Science, Brain Research Institute,
University of Niigata, Niigata, Japan

[2] Administrative Section of Radiation Protection, National Institute of Neuroscience,
National Center of Neurology and Psychiatry, Japan

[3] Department of Neurological Science, Graduate School of Medicine,
Nippon Medical School, Tokyo, Japan

[4] Department of Neurology, University of California Davis, Davis, California, USA

Synthesis of TGF-019

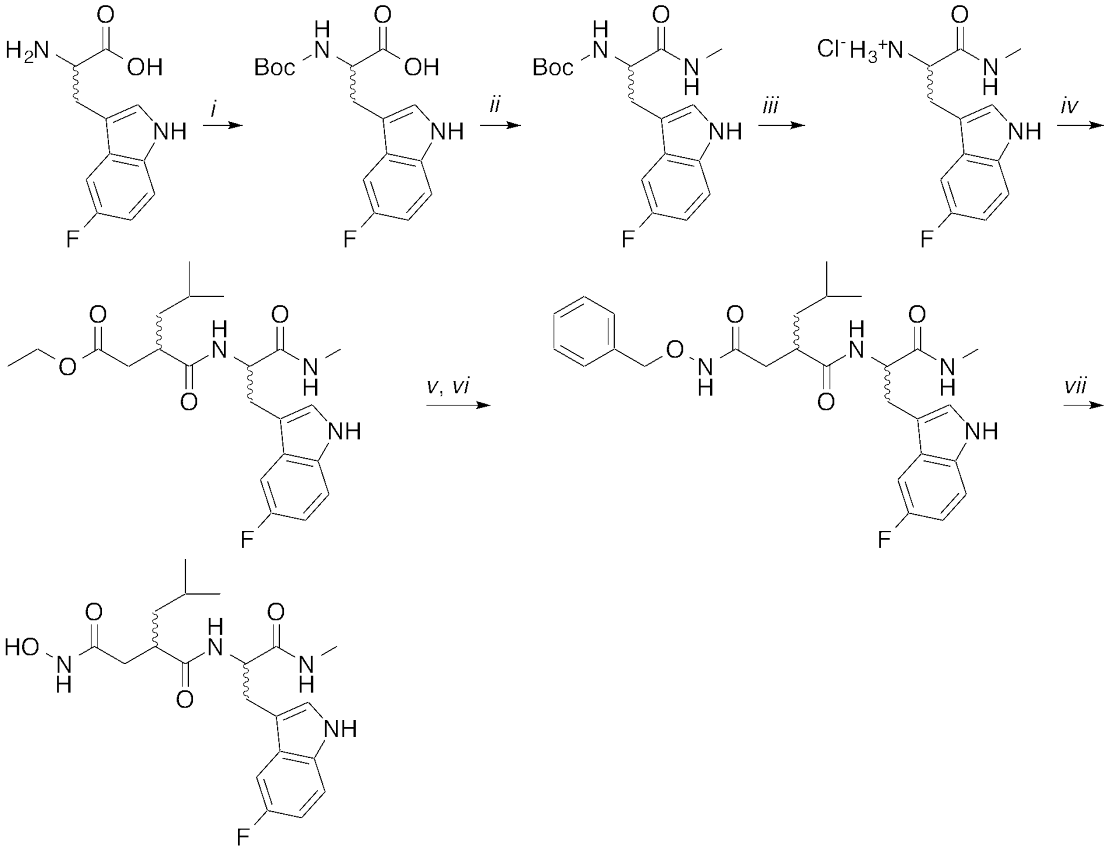

TGF-019

Figure S-1. Synthetic scheme for TGF-019. (*i*) $(Boc)_2O$, NaOH, H_2O, rt, 48 h. (*ii*) NH_2CH_3•HCl, BOP, TEA, DMF, rt, 24 h. (*iii*) 4M HCl/dioxane, dioxane, rt, 14 h; Et_2O. (*iv*) (Rac)-2-[(Ethoxycarbonyl)methyl]-4-methylpentanoic acid, EDC, HOBt•H_2O, NMM, DMF, rt, 24 h. (*v*) LiOH, 67% EtOH, rt, 24 h; HCl. (*vi*) O-benzylhydroxylammonium hydrochloride, BOP, NMM, DMF, rt, 16 h. (*vii*) NH_4HCO_2, 10% Pd-C, MeOH, rt, 27 h.

General methods: (Rac)-2-[(Ethoxycarbonyl)methyl]-4-methylpentanoic acid was obtained from American Biochemicals (College Station, TX, USA), and was used as received. Additional reagents were sourced from Sigma-Aldrich (Tokyo, Japan), Wako Pure Chemical Industries (Osaka, Japan), TCI (Tokyo, Japan) or Nacalai Tesque (Kyoto, Japan) at the highest grade possible, and were used as received. Analytical thin-layer chromatography (TLC) was performed using Sigma-Aldrich F254 indicating TLC plates, 200 μM thickness, polyester backing, which were visualized under UV light unless otherwise noted. Preparative thin layer chromatography (TLC) was performed using Analtech F254 indicating preparative TLC plates (1000 μm). Preparative flash chromatography was performed using Wako-gel 300 silica gel. ^{1}H nuclear magnetic resonance (^{1}H-NMR) spectra were recorded at 300 MHz on a Varian (Varian Inc, Palo Alto, CA, USA) Mercury 300 spectrometer and are reported in parts per million (PPM) downfield from an internal tetramethylsilane (TMS) peak, unless otherwise noted. Peaks attributable to individual diasteromers are indicated where possible. Analytical ultra-performance liquid chromatography (UPLC) and high-resolution mass spectroscopy (HR-MS) were performed on a Waters (Milford, MA, USA) Acquity UPLC combined with a Waters LCT Premier XE mass detector. Additional UPLC analytical data was obtained using Waters Acquity UPLC PDA and ELS detectors. Analytical measurements were performed using Acquity UPLC BEH C18 1.7 μm 2.1 x 50 mm columns (Waters) eluted with a 90-10% gradient of water/acetonitrile. Mass spectra were recorded in high-resolution mode unless otherwise noted. Exact mass formulae and isotopic distribution correlations were done using ISOMABS (version 5a, Trace Analysis Research Center, Dalhousie University, NS, Canada). Systematic names for each compound were generated from the 2-D structure using ACD/I-Lab (Advanced Chemistry Development Inc, https://ilab.acdlabs.com/iLab2/index.php).

(±)-*N*-(*tert*-butoxycarbonyl)-5-fluorotryptophan (Boc-(5F)Trp-OH):

Boc_2O (0.490 g, 2.25 mmol) was added to a solution of (±)-(5-fluoro)trpytophan (0.500 g, 2.25 mmol) and NaOH (0.213 g, 5.06 mmol) in deionized water (5 mL). The resulting mixture was stirred at room temperature for 48 h. The clear solution was then washed with diethyl ether (10 mL), and acidified with 6 M HCl (1.66 mL). The resulting mixture was extracted with ethyl acetate (2 x 15 mL), and the combined extracts were dried over $MgSO_4$, filtered and evaporated to give a white solid, 0.580 g (80%). NMR was consistent with that reported for commercial products, and was used as obtained.

^{1}H NMR ($CDCl_3$): δ1.44 (s, 9H), 3.20-3.36 (m, 4H), 4.58-4.67 (br, 1H), 4.98-5.09 (br, 1H), 6.95 (dt, J_1 = 9 Hz, J_2 = 2.4 Hz, 1H), 7.10 (d, J = 2.4 Hz, 1H), 7.22 (d, J = 2.4 Hz, 1H), 7.29 (d, J = 4.5 Hz, 1H), 8.10 (br-s, 2H).

(±)-*N*α-(*tert*-butoxycarbonyl)-5-fluoro-*N*-methyltryptophanamide (Boc-(5F)Trp-NHCH$_3$):

Solid Boc-(5F)Trp-OH (0.579 g, 1.80 mmol) was added to methylamine hydrochloride (0.245 g, 3.60 mmol) followed by BOP (0.796 g, 1.80 mmol), to which DMF (10 mL) was added. Triethylamine (0.879 mL, 5.10 mmol) was then added and resulting solution was stirred at room temperature for 24 h under Ar. At which time, the solvent was removed under reduced pressure. The resulting oily residue was partitioned between EtOAc (15 mL) and 10% citric acid (10 mL). The organic layer was separated, and subsequently washed with additional 10% citric acid (10 mL), brine (10 mL) and saturated aqueous $NaHCO_3$ (1x20 mL). The organic solution was dried over $MgSO_4$, filtered and evaporated to give a white solid, which was washed with ether (10 mL), and dried in vacuo to give a white solid. ^{1}H NMR and UPLC-MS spectra were consistent with the assigned product, purity (DAD) >95%. Material used without further purification, 0.485 g (71%).

^{1}H NMR ($CDCl_3$): δ1.42 (s, 9H), 2.70 (d, J = 4.8 Hz, 3H), 3.08-3.29 (m, 2H), 3.37 (br-q, J_1 = 13.5 Hz, J_2 = 7.5 Hz, 1H), 5.07 (br-s, 1H), 5.76 (br-s, 1H), 6.95 (td, J_1 = 9.0 Hz, J_2 = 2.4 Hz, 1H), 7.10

(d, J = 2.4 Hz, 1H), 7.26-7.31 (m, 2H), 8.11 (br-s, 1H). UPLC: rt = 1.54 min. HR-MS: mass calculated for $C_{17}H_{23}N_3O_3F$ ($M+H^+$), m/z = 336.1718; found 336.1518.

(±)-5-Fluoro-*N*-methyltryptophanamide hydrochloride (H-(5F)Trp-$NHCH_3$):

Boc-(5f)Trp-$NHCH_3$ (1.17 g, 3.48 g) was suspended in dry dioxane (10 mL). The reaction vessel was blanked with Ar and sealed by a septum, through which 4 M HCl-dioxane (5 mL, 20 mmol) was added by syringe. The resulting solution was stirred at room temperature for 14 h under Ar. Following which, dry ether (25 mL) was added. The supernate was decanted and the solid reside was washed with additional chilled ether (2 x 10 mL). The solid was then dissolved in deionized water (15 mL), and the aqueous solution washed with hexanes (15 mL). The aqueous solution was dried under reduced pressure, and the residue was lyophilized from ultra-pure water to give a tan colored solid, which darkened upon standing. The crude product was used immediately as obtained, 926 mg (98%).

Ethyl 3-{[3-(5-fluoro-1*H*-indol-3-yl)-1-(methylamino)-1-oxopropan-2-yl]carbamoyl}-5-methylhexanoate (pro-EtO-TGF-019):

(Rac)-2-[(Ethoxycarbonyl)methyl]-4-methylpentanoic acid (0.294 g, 1.48 mmol) was added to HOBt•H_2O (0.226 g, 1.48 mmol), to which DMF (10 mL) was added. To this solution, EDC (0.261 g, 1.48 mmol) and NMM (0.482 mL, 4.44 mmol) were then added, followed by an additional aliquot of DMF (5 mL). The resulting solution was stirred for 24 h at room temperature. At which time, the solvent was removed under reduced pressure and the resulting residue was partitioned between EtOAc (20 mL) and 10% citric acid (20 mL). The organic solution was subsequently washed with additional 10% citric acid (20 mL), brine (20 mL), sat. $NaHCO_3$ (2 x 20 mL) and brine (20 mL), then dried over $MgSO_4$, filtered and evaporated under reduced pressure to give a yellow foam, 0.432 g. The thusly obtained residue was purified by flash chromatography, Wako-gel 300, eluted with 50% EtOAc/Hex) to give a colorless foamy solid. ^1H NMR was consistent with the expected product as mixture of diasteromers, although its assignment was complicated by the presence of multiple diastereomers and slow interconversion of rotational isomers. UPLC-MS was constant with the assigned structure as a diasterotopic mixture. Total chemical purity > 95% (DAD), 0.332 g (54%).

^1H NMR (DMSO-D_6): δ0.57-1.15 (m, 12H), 2.19-2.46 (m, 1H), 2.54-2.62 (m, 3H [minor diasteromer: δ2.55, d, J = 4.6 Hz; major diasteromer: δ2.59, d, J = 4.6 Hz]), 2.72-2.87 (m, 2H), 2.98-3.20 (m, 2H), 4.00 (br-q, J_1 = 9 Hz, J_2 = 7.2 Hz, 2H), 4.38-4.47 (m, 1H), 6.87 (br-t, J = 9.6 Hz), 7.06-7.42 (m, 2H), 7.70-7.93 (m, 1H), 8.06-8.23 (m, 1H). UPLC: major diasteromer, rt = 1.66 min (75%); minor diasteromer, rt = 1.72 min (25%). HR-MS mass calculated for $C_{22}H_{31}N_3O_4F$ ($M+H^+$), m/z = 420.2293; found 420.2402.

3-{[3-(5-Fluoro-1*H*-indol-3-yl)-1-(methylamino)-1-oxopropan-2-yl]carbamoyl}-5-methylhexanoic acid (pro-OH-TGF-019):

Ethyl 3-{[3-(5-fluoro-1*H*-indol-3-yl)-1-(methylamino)-1-oxopropan-2-yl]carbamoyl}-5-methylhexanoate (0.121 g, 0.29 mmol) was dissolved in ethanol (6.6 mL) to which LiOH (0.0365 g, 0.870 mmol) was added, followed by deionized water (3.3 mL). The resulting solution was stirred for 24 h, which was then concentrated under reduced pressure. The residue was redissolved in deionized water (20 mL) to which 1M HCl (2 mL) was added dropwise with stirring. The resulting solid was triturated, then partitioned between EtOAc (10 mL) and brine (10 mL). The organic phase was separated, dried over $MgSO_4$, filtered and evaporated to give a pale white solid, which visibly yellowed upon standing, and was immediately carried onto the following step, 0.103 g (91%)

***N*^{4}-benzyloxy-*N*^{1}-[3-(3-fluoro-1*H*-indol-3-yl)-1-(methylamino)-1-oxopropan-2-yl]-2-(2-methylpropyl)butanediamide (BnO-TGF-019):**

O-benzylhydroxylammonium chloride (0.125 g, 0.780 mmol) was added to 3-{[3-(5-Fluoro-1*H*-indol-3-yl)-1-(methylamino)-1-oxopropan-2-yl]carbamoyl}-5-methylhexanoic acid (0.152 g, 0.390) followed by DMF (5 mL). NMM (0.190 mL, 1.76 mmol) was added to the resulting solution followed by BOP (0.173 g, 0.390 mmol). The resulting solution was stirred for 16 h at room temperature under Ar. At which time the solvent was removed under reduced pressure. The residue was partitioned between EtOAc (10 mL) and 10% citric acid (10 mL). The organic phase was separated and washed with brine (10 mL) and saturated aqueous $NaHCO_3$ (2 x 10 mL), then dried over $MgSO_4$, filtered and evaporated under reduced pressure to give a colorless semi-solid, 0.196 g. The crude reside was purified by preparative TLC (10% MeOH/DCM) to give a colorless foamy solid. ^1H NMR was consistent with the assigned structure as mixture of diasteromers, although its assignment was complicated by the presence of multiple diastereomers and slow interconversion of rotational isomers. UPLC-MS was consistent with the assigned structure, although the diasteromer peaks were insufficiently resolved to determine their ratio. Total chemical purity > 95% (DAD), 0.136 g (70%).

^1H NMR (DMSO-D_6): δ0.52-0.83 (m, 7H), 0.95-1.40 (m, 2H), 1.91-2.21 (m, 2H), 2.53-2.62 (m, 2H), 2.71-3.00 (m, 4H), 4.39-4.46 (m, 1H), 4.71-4.76 (m, 2H), 6.87 (dt, J_1 = 9 Hz, J_2 = 2.4 Hz, 1H), 7.17-7.20 (m, 1H), 7.26-7.37 (m, 7H) 7.86-7.93 (m, 1H), 8.01-8.26 (m, 1H) 10.90 (br-s, 1H), 10.99-11.13 (br-m, 1H). ULPC: rt = 1.65 min, diasteromer peaks unresolved. HR-MS: mass calculated for $C_{27}H_{34}N_3O_4F^+$ (M+H$^+$), m/z = 497.2559; found 497.2643.

N^1-[3-(5-fluoro-1*H*-indol-3-yl)-1-(methylamino)-1-oxopropan-2-yl]-N^4-hydroxy-2-(2-methylpropyl)butanediamide (TGF-019):

N^4-benzyloxy-N^1-[3-(3-fluoro-1*H*-indol-3-yl)-1-(methylamino)-1-oxopropan-2-yl]-2-(2-methylpropyl)butanediamide (0.133 g, 0.268 mmol) was dissolved in MeOH (5 mL) to which ammonium formate (0.168 g, 2.68 mmol) was added, followed by 10% Pd-C (15 mg) and additional MeOH (2 mL). The reaction vessel was evacuated and charged with Ar, and the resulting mixture was stirred at room temperature for 27 h. Following which, the mixture was filtered through a pad of celite, which was subsequently washed with additional MeOH (20 mL). The combined filtrate and washings were evaporated to give a yellow residue, which was partitioned between 0.1 M HCl (20 mL) and DCM (10 mL). The organic phase was separated, and the aqueous phase re-extracted with EtOAc (2 x 10 mL). The combined EtOAc extracts were dried over Na_2SO_4, filtered and evaporated to give a white solid. ^1H NMR was consistent with the expected structure as mixture of diasteromers, although its assignment was complicated by the presence of multiple diastereomers and slow interconversion of rotational isomers. UPLC-MS was consistent with the assigned structure as a 2.4:1 mixture of diasteromers. Total chemical purity = 95% (DAD), 0.075 g (69%).

^1H NMR (DMSO-D_6): δ0.52-0.85 (m, 7H), 0.97-1.44 (m, 2H), 1.88-2.19 (m, 2H), 2.54-2.64 (m, 2H), 2.71-3.08 (m, 4H), 4.37-4.42 (m, 0.7H), 4.86-4.89 (br, 0.3H), 6.82-6.91 (m, 1H), 7.15-7.37 (m, 3H), 7.84-8.01 (m, 1H), 8.23-8.45 (m, 1H), 8.69-8.78 (m, 1H), 10.36-10.49 (br-m, 1H), 10.90 (br-s, 1H). ULPC: diasteromer 1, rt = 1.25 min (32%); diasteromer 2, rt = 1.31 min (68%). HR-MS: mass calculated for $C_{20}H_{28}FN_4O_4$ (M+H$^+$), m/z = 407.2089; found, 407.2074.

2-(4-hydroxamato-2-isobutylsuccinamido)-3-(5-fluoroindo-3-yl)propiomethylamide sodium salt (TGF-019-Na$^+$):

TGF-019 (0.070 g, 0.172 mmol) was suspended in deionized water (5 mL) to which 1 M NaOH (0.189 mL, 0.189 mmol) was added with stirring. The resulting clear solution was filtered through a 20 µM ultrafiltration membrane then lyophilized to give a white solid, which was used as obtained, 0.065 g.

Abbreviations:

BOP,	(Benzotriazol-1-yloxy)tris(dimethylamino)phosphonium hexafluorophosphate
DCM,	Dichloromethane
DMF,	N,N-Dimethylformamide
EDC,	3-(Ethyliminomethyleneamino)-*N*,*N*-dimethylpropan-1-amine hydrochloride
EtOAc,	Ethylacetate
EtOH,	Ethanol
MeOH,	Methanol
NMM,	4-Methylmorpholine
TEA,	Triethylamine

***In vitro* MMP inhibition**

The ability of TGF-019 to interact with MMP2, MMP3 and MMP9 was investigated using an *in vitro* inhibition assay. The assay was outsourced to Eurofins Panlabs Discovery Services Taiwan (Taipei, Taiwan), assay numbers 114210, 114310 and 114910, respectively for MMP2, MMP3 and MMP9, in a compound-blinded fashion. TGF-019 were screened according to the following general procedure:[1,2] Human recombinant MMP proenzyme (MMP2, MMP3 or MMP4, respectively) was activated with APMA for 60 minutes at 37 °C. Test compound and/or vehicle was pre-incubated with the activated enzyme in modified MOPS buffer (pH 7.2) for 60 min at 37 °C. The reaction is initiated by addition of 4 mM Mca-Pro-Leu-Gly-Leu-Dap-Ala-Arg for another 120 min incubation period. Determination of the amount of Mca-Pro-Leu-Gly formed is read spectrofluorimetrically at 340 nm/400 nm. TGF-019 screened at 1 mM for n = 3 replications.

MMP2: TGF-019 %INH = 94.7 ± 0.4 %
MMP3: TGF-019 %INH = 52.4 ± 0.3 %
MMP9: TGF-019 %INH = 104.0 ± 0.2 %

References

1. Knight CG, Willenbrock F, Murphy G. A novel coumarin-labelled peptide for sensitive continuous assays of the matrix metalloproteinases. FEBS Lett. 1992;296:263-266.
2. Olson MW, Gervasi DC, Mobashery S, Fridman R. Kinetic analysis of the binding of human matrix metalloproteinase-2 and -9 to tissue inhibitor of metalloproteinase (TIMP)-1 and TIMP-2. J Biol Chem. 1997;272:29975-29983.
